# TDP-43-Mediated Toxicity in HEK293T Cells: A Fast and Reproducible Protocol To Be Employed in the Search of New Therapeutic Options against Amyotrophic Lateral Sclerosis

**DOI:** 10.3390/cells9010068

**Published:** 2019-12-26

**Authors:** Débora Lanznaster, Jérôme Bourgeais, Clement Bruno, Rudolf C. Hergesheimer, Rose-Anne Thepault, Patrick Vourc’h, Philippe Corcia, Christian R. Andres, Olivier Herault, Hélène Blasco

**Affiliations:** 1 UMR 1253, iBrain, University of Tours, Inserm, 37000 Tours, Francehelene.blasco@univ-tours.fr (H.B.); 2 CNRS ERL7001, EA 7501 GICC, University of Tours, 37000 Tours, Franceolivier.herault@univ-tours.fr (O.H.); 3 CHU de Tours, Service de Biochimie et Biologie Moléculaire, 37000 Tours, France

**Keywords:** ALS, TDP-43, aggregation, cellular death, apoptosis, metabolomics

## Abstract

Cytoplasmic TDP-43 aggregates are a hallmark of amyotrophic lateral sclerosis (ALS). Today, only two drugs are available for ALS treatment, and their modest effect prompts researchers to search for new therapeutic options. TDP-43 represents one of the most promising targets for therapeutic intervention, but reliable and reproducible in vitro protocols for TDP-43-mediated toxicity are lacking. Here, we used HEK293T cells transfected with increasing concentrations of TDP-43-expressing plasmid to evaluate different parameters of toxicity and alterations in cellular metabolism. Overexpression of TDP-43 induced aggregates occurrence followed by the detection of 25- and 35-kDa forms of TDP-43. TDP-43 overexpression decreased cell viability and increased cells arrested at G2/M phase and nuclear fragmentation. Analysis of the energetic metabolism showed a tendency to decrease oxidative phosphorylation and increase glycolysis, but no statistical differences were observed. Metabolomics revealed alterations in different metabolites (mainly sphingolipids and glycerophospholipids) in cells overexpressing TDP-43. Our data reveal the main role of TDP-43 aggregation in cellular death and highlight novel insight into the mechanism of cellular toxicity induced by TDP-43. Here, we provide a simple, sensitive, and reliable protocol in a human-derived cell line to be used in high-throughput screenings of potential therapeutic molecules for ALS treatment.

## 1. Introduction

Transactive Response DNA Binding Protein 43 (TDP-43) is a ubiquitous 43 kDa-protein implicated in multiple steps of transcriptional and post-transcriptional regulation. Under physiological conditions, the majority of TDP-43 is localized in the nucleus, where it controls mRNA stability, translation, and nucleocytoplasmic transport promoted by a small proportion of TDP-43 that continuously shuttles between the nucleus and cytoplasm [1,2].

Full-length TDP-43 protein is found in cytoplasmic aggregates in degenerated motor neurons and surrounding glial cells in the central nervous system of patients suffering from amyotrophic lateral sclerosis (ALS). Currently, these aggregates are considered the hallmark of ALS. It remains controversial whether TDP-43 aggregation is the principal cause of the motor neuronal degeneration observed in ALS patients, but recent studies shed a light into the key role of TDP-43 aggregates in causing the observed neurodegeneration. Further, TDP-43 aggregates are present in patients with and without any mutations in the TDP-43 encoding gene, *TARDPB*, and in patients with mutations in other genes involved in ALS pathology, like *SOD-1* and *C9orf72* (reviewed by [3]).

Similar pathophysiological mechanisms are described for both genetic and sporadic ALS patients, and as 97% of ALS patients present TDP-43 aggregation, it is plausible to suggest a link between TDP-43 and some of the pathogenic mechanisms [4,5]. Data published from cellular and animal models of ALS based on TDP-43 toxicity focused on mutant forms of TDP-43 protein, or even smaller toxic species derived from TDP-43 full-length protein, as 25- and 35-kDa fragments found in ALS patients. During the process of selection of drug candidates, we must determine the most promising ones based on objective and reliable criteria to move with on the preclinical steps with an optimized number of candidates. In the present study, we deepen the findings about wild-type, full-length TDP-43-mediated toxicity by exploring different parameters of cellular toxicity and alterations in the metabolic status of the cell. Our project aims to validate the most relevant parameters associated with TDP-43 aggregation, providing a suitable protocol applied to evaluate neuroprotective effects of new potential therapeutics against ALS. We suggest that these parameters may be also useful in animal models and in patients as markers of drug engagement or response to new therapeutics.

## 2. Materials and Methods

### 2.1. Plasmids

TDP-43-bearing plasmids consisted of human TDP-43. For visualization of TDP-43 expression, the cDNA insert was cloned into pcDNA6.2 N-EmGFP vector (N-terminal GFP) with six histidine residues (6xHis) added to the C terminus of TDP-43. For all the other experiments, TDP-43-6xHis cDNA was cloned into pcDNA3.3 vector (the 6xHis were fused to TDP-43 with the purpose of purification of TDP-43 protein for drug screening protocols). pcDNA6.2 N-EmGFP-6xHis and pcDNA3.3 vectors were used in control conditions.

### 2.2. Cells

HEK293T cells (American Type Culture Collection, U.S.A.) were grown in Dulbecco’s modified Eagle’s medium (DMEM) supplemented with 5% (*v*/*v*) fetal bovine serum (FBS) at 37 °C and in an atmosphere of 5% CO_2_. Cells were plated in six-well dishes at a concentration of 2.5 × 10^5^ cells/well and cultured overnight before transfection. Cells were transiently transfected using jetPEI reagent (Polyplus-transfection^®^ SA; ratio cDNA 1:2 jetPEI according to the manufacturer instructions). Cells were transfected with increasing concentrations of TDP-43-containing plasmids (1, 2, 3, or 4 µg). Control plasmids were transfected at the highest concentration (4 µg). Assays were realized 48 h after transfection.

### 2.3. Western Blot

1 × 10^6^ HEK293T cells were harvested in ice-cold phosphate-buffered saline (PBS) and centrifuged at 900× *g*. Cell lysis was induced with ice-cold Pierce-RIPA buffer containing protease inhibitors cocktail (ThermoFisher Scientific) and Benzonase nuclease (Sigma-Aldrich; 25 U/1 mL of buffer). Cells were centrifuged at 5000× *g*, and the half of the supernatant was collected as crude extract. Cells were centrifuged at 15,000× *g* for collection of the soluble fraction (supernatant) and insoluble fraction (pellet resuspended in RIPA/Urea 6M). Protein content was measured by Lowry method (Bio-Rad). Proteins were separated in 4–20% SDS-PAGE gel (Bio-Rad) and transferred to PVDF membranes. After blocking with 5% milk in TBS-T buffer, membranes were incubated overnight with an antibody anti-TDP-43 C-terminus (polyclonal rabbit, 1:5000; ProteinTech) and anti-p38 MAPK or anti-phospho p38 MAPK (Thr180/Thr182; polyclonal rabbit, 1:5000; Cell Signaling), followed by 1 h incubation with a secondary antibody coupled to a horseradish peroxidase (HRP; anti-rabbit, 1:5000). Chemiluminescence was observed using Chemidoc (Bio-Rad) after incubation with ECL. Bands intensity was measured with Image Lab software (Bio-Rad). Actin (polyclonal anti-mouse HRP-conjugated, 1:100,000) was used as internal control.

### 2.4. Cell Viability Assays

After 48 h of transfection, cells were incubated in 0.5 mg/mL 3-(4,5-dimethylthiazol-2-yl)-2,5-diphenyltetrazolium bromide (MTT) (Sigma-Aldrich) for 30 min at 37 °C. The tetrazolium ring of MTT can be cleaved by active dehydrogenases in order to produce a precipitated formazan. The medium was withdrawn, the precipitated formazan was solubilized with 500 μL of dimethyl sulfoxide (DMSO), and cellular viability was quantified by spectrophotometry at a wavelength of 570 nm.

For the quantification of live cells (measurement of propidium iodide (PI) incorporation (Sigma Aldrich) and trypan blue exclusion test), cells were washed with PBS and incubated with Trypsin (Gibco) for 5 min at 37 °C and centrifuged at 900× *g*. Cells were then resuspended in 1 mL PBS with 1% FBS. 10 µL were used to test for trypan blue 0.4% exclusion (Life technologies, according to manufacturer instructions). 0.3 × 10^6^ cells were incubated with 10 µg/mL PI in PBS with 1% FBS for 30 min at 37 °C. Cells were then analyzed by flow cytometry on a Becton Dickinson Accuri™ C6 Plus flow cytometer, and results are presented as the percentage of positive cells (% Pos) in relation to the control group (cells transfected with the empty pcDNA3.3 vector).

Lactate dehydrogenase (LDH) activity was measured in the medium of the different transfection conditions. Briefly, the medium (2 mL) was collected from the wells, centrifuged at 900× *g* for 5 min to pellet any cell debris, and frozen at −20 °C until analysis. Absorbance was measured in the Roche/Hitachi cobas^®^ according to the manufacturer’s instructions.

### 2.5. Cell Cycle Analysis

Cell cycle analysis was performed with the BD Cycletest Plus DNA kit (BD Biosciences), according to the manufacturer’s instructions. Briefly, 10^6^ HEK293T cells were washed, permeabilized, and stained with PI after RNA elimination. Samples were immediately analyzed by flow cytometry (Becton Dickinson Accuri™ C6 flow cytometer). At least 50,000 events were collected for each condition. Cells in phases G0/G_1_, S, and G_2_/M, as well as Sub-G_1_ events (which comprise all fragmented nuclei, including apoptotic bodies), were analyzed as described before [6].

### 2.6. Analysis of Reactive Oxygen Species (ROS) Levels

Intracellular reactive oxygen species (ROS) levels were measured using 5- (and-6-)chloromethyl-2′,7′-dichlorodihydrofluorescein diacetate, acetyl ester (CM-H_2_DCFDA) (Invitrogen, Carlsbad, CA, USA). Cells (0.3 × 10^6^) were washed with PBS with 1% FBS and stained with 10 μM CM-H2DCFDA for 30 min at 37 °C. Cells were then analyzed by flow cytometry (Becton Dickinson Accuri™ C6 flow cytometer).

### 2.7. Analysis of Mitochondrial Dysfunction

Tetramethylrhodamine methyl ester (TMRM, Invitrogen) is a cell-permeant probe that accumulates in mitochondria with intact membrane potentials. Cells (0.3 × 10^6^) were washed with PBS with 1% FBS and stained with 20 nM TMRM for 30 min at 37 °C. Cells were analyzed by flow cytometry (Becton Dickinson Accuri™ C6 flow cytometer).

### 2.8. Energy Metabolism Analyses

The cellular oxygen consumption rate (OCR) and extracellular acidification rate (ECAR) were determined using a Seahorse™ XF96 Flux analyzer (Seahorse Bioscience, North Billerica, MA, USA). Experiments were performed according to the manufacturer’s instructions. Briefly, HEK293T cells were seeded in XF96 cell culture plates at 10^4^ cells per well and transfected with increasing concentrations of TDP-43–6xHis plasmid. The XF96 sensor cartridges were hydrated with 200 μL calibrant pH 7.4 and stored overnight at 37 °C without CO_2_. On the day of analysis, the culture medium was replaced with XF DMEM medium, lacking bicarbonate (pH 7.4), and supplemented with glutamine (2 mM). Cells were then incubated at 37 °C in a non-CO_2_ incubator for 1 h. Sequential injection of glucose (10 mM), oligomycin (1 μM), dinitrophenol (DNP, 100 μM), or rotenone/antimycin A (0.5 mM) were added according to the supplier’s technical specifications to determine the principal metabolic parameters (Sigma-Aldrich). Specific mitochondrial and glycolytic ATP productions were assessed using Seahorse XF Real-Time ATP Rate Assay Kit (Seahorse Bioscience) according to the manufacturer protocol. Results were normalized in relation to DNA content per well using CyQuant dye (ThermoFisher Inc.).

### 2.9. Metabolomics Analysis

A targeted, quantitative approach was used based on the AbsolutIDQ™ p180 kit (Biocrates, Innsbruck, Austria) using a Flow Injection Analysis and High-Performance Liquid Chromatography (HPLC-) Mass Spectrometry (MS/MS) assay. This assay kit enables the quantification of 188 metabolites [7]. Cell lysates were obtained from 3 × 10^6^ cells resuspended in ice-cold ethanol/0.01M PBS subjected to freeze/thaw cycles. Samples were loaded onto a filter paper and dried in a stream of nitrogen for derivation with a solution of phenyl-isothiocyanate 5%. Subsequently, dried residues were extracted with methanol containing 5 mM ammonium acetate. The MetIDQ^®^ software (Biocrates) is used to calculate the concentrations of individual metabolites. Quality controls (QC) were analyzed regularly on the plate (every 8 samples) to ensure the stability of the mass system over time.

### 2.10. Statistical Analysis

For metabolomics analysis, both multivariate and univariate analyses were performed using MetaboAnalyst version 4.0 (http://www.metaboanalyst.ca). The classification method was based on Partial Least-Squares Discriminant Analysis (PLS-DA). Values of variable importance in projection [8] represent the importance of the metabolite for the PLS-DA models. The score scatter plots show the classified samples and the loadings plot characterize the relation between the Y and X variables. The univariate analysis of metabolites levels was based on the fold-change values and the threshold of significance after nonparametric Wilcoxon test. The metabolites that had ‘variable importance in the projection’ (VIP) values > 1 and were determined by a fold-change > 2 (and this for each comparison) were retained as the most relevant for further discussion [9,10].

Results are shown as mean ± standard error of the mean (SEM). Cytometry results were analyzed using FlowJo VX software, and representative graphs from all analyses are shown in Figure S1. Kruskal-Wallis test followed by Dunn’s multiple comparison test (when relevant) was performed using GraphPad Prism 7.

## 3. Results

### 3.1. Cellular Distribution and Aggregation of TDP-43

**Figure 1:**
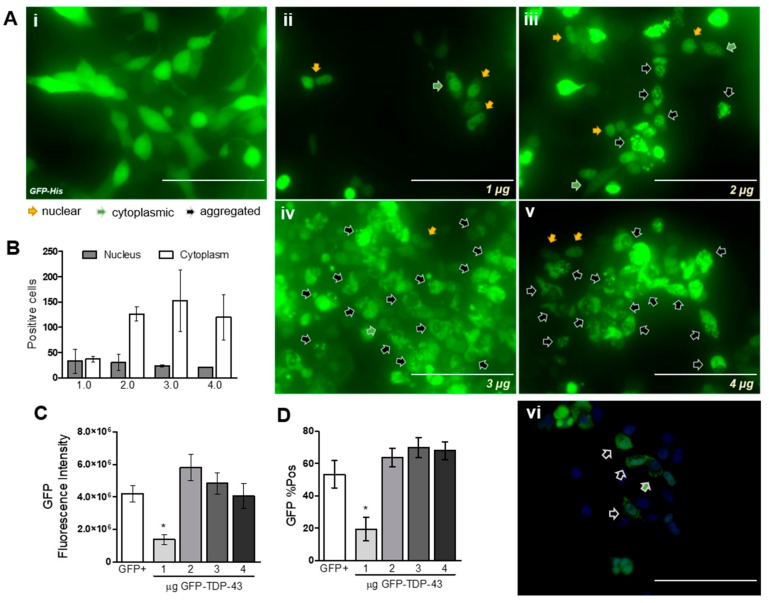
HEK293T transfection with increasing quantities of GFP-TDP-43 induces an increase in TDP-43 expression and cytoplasmic aggregation. (**A**) i Control cells transfected only with GFP (4 µg) show presence of GFP signal in the whole cell. (**A**) ii–v Increasing concentrations of GFP-TDP-43 showing different cellular distributions (observed in detail in (**A**) vi; scale bar = 100 µm). Cell nucleus was counterstained with DAPI (2 μg/mL; Sigma–Aldrich). Scales bar = 200 µm for (**A**) i–v. (**B**) The increase in expression was followed by an increase in the number of cells presenting cytoplasmic aggregates (*n* = 2). (**C**) and (**D**) Increase in the number of positive cells and median fluorescence signal for GFP analyzed by flow cytometry (*n* = 5). Nonparametric Kruskal–Wallis test revealed * *p* < 0.05 for TDP-43 1 μg when compared to GFP+ group (positive control: cells transfected with 4 μg of GFP plasmid).

HEK293T cells transfected with increasing quantities of TDP-43-encoding plasmid showed an expected increase in the number of positive cells expressing GFP-fused TDP-43. As can be observed in Figure 1Ai, it is possible to observe GFP expression in the whole cell, along with normal cell morphology. However, cells transfected with increasing concentrations of GFP-TDP-43 plasmid showed different cellular distributions of the fluorescent signal (Figure 1Aii–v): GFP signal was observed in the nucleus (orange arrows), the cytoplasm (green arrows), or as aggregates (black arrows). The quantification of GFP-positive cells demonstrates an increase in TDP-43 localization in the cytoplasm with the higher plasmid concentrations (Figure 1B). The different cellular distributions can be better observed in Figure 1Avi, together with the cytoplasmic aggregation of TDP-43. Importantly, the vast majority of cells expressing GFP-TDP-43 proteins lost their normal cellular morphology and present a round shape. Flow cytometry analysis revealed no statistical differences in the quantity of positive cells between GFP cells and GFP-TDP-43 2 µg, 3 µg, and 4 µg (Figure 1C). The increase in GFP-TDP-43 expression was also followed by an increase in the GFP signal in each cell analyzed by flow cytometry (Figure 1D).

For the following experiments, we turned to a plasmid containing only TDP-43-6xHis, as GFP-induced expression was toxic in preliminary experiments (Figures S2 and S3; also discussed elsewhere [11,12]).

**Figure 2:**
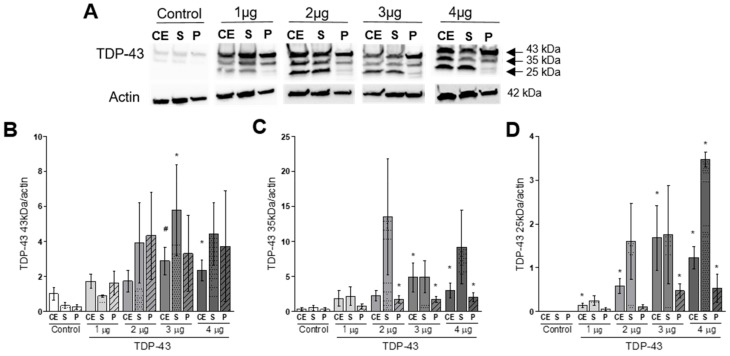
TDP-43 overexpression increases TDP-43 protein full-length (43kDa) presence in soluble and insoluble fractions of cells lysates (**B**) together with the detection of 35- (**C**) and 25-kDa (**D**) C-terminal fragments (CTFs). Representative Western blots are shown in panel (**A**). CE: crude extract; S: soluble fraction; P: insoluble fraction. Nonparametric Kruskal–Wallis test revealed * *p* < 0.05 and # *p* = 0.053 compared to the respective control groups (CE x CE; S x S; P x P; *n* = 3–6).

Western blot analysis confirmed that TDP-43 transfection also led to an increase in the total TDP-43 protein expressed by HEK293T cells (Figure 2A,B). Interestingly, TDP-43 transfection induced increase in C-terminal fragments (CTFs) of TDP-43 of 25- and 35-kDa (Figure 2C,D). The increase in TDP-43 proteins full length and fragments was also observed in soluble (S) and insoluble (P) cellular fractions, meaning that both soluble and aggregated forms of TDP-43 were found only in transfected cells and increased in relation to the concentration of plasmid used.

### 3.2. Cell Viability Assays

**Figure 3:**
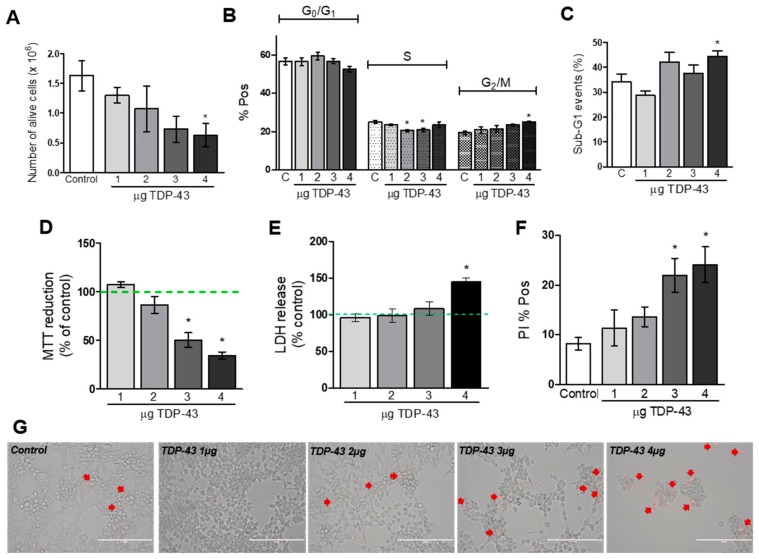
TDP-43 overexpression induces a decrease in cellular viability measured after 48 h of transfection. (**A**): Number of alive cells analyzed by trypan blue exclusion (*n* = 3; * *p* < 0.05). (**B**) Cell cycle analysis performed by flow cytometry (*n* = 4–5; * *p* < 0.05). (**C**) Percentage of sub-G1 events (fragmented nuclei) analyzed by flow cytometry (*n* = 4–5; * *p* < 0.05). (**D**) Cell viability analyzed by MTT reduction (*n* = 4; * *p* < 0.05). (**E**) LDH released in the medium (*n* = 4; * *p* < 0.05). (**F**) Percentage of positive cells for PI fluorescence (*n* = 5–6; * *p* < 0.05). (**G**) Microscopic analysis of cells transfected with increasing concentrations of TDP-43 and incubated with PI (scale bar = 200 µm).

After 48 h of transfection, the total number of alive cells (measured with the trypan blue exclusion test) was significantly smaller in cells transfected with increasing concentrations of TDP-43 compared to control cells (*p* < 0.05 for TDP-43 4 µg compared to cells transfected with 4 µg of empty plasmid; Figure 3A). Cell cycle analysis showed that the percentage of cells arrested at the G2/M phase was increased when cells were transfected with TDP-43 4 µg (Figure 3B). Intriguingly, TDP-43 at concentrations of 2 and 3 µg induced a decrease in the percentage of cells in S phase (Figure 3B). TDP-43 4 µg also increased the number of fragmented nuclei (sub-G1 events; Figure 3C). The same trend was observed in relation to the MTT reduction. Increasing concentrations of TDP-43 induced a concentration-related decrease in cell viability (*p* < 0.05 compared to control group; Figure 3D). To confirm the decrease in cell viability induced by TDP-43, we analyzed the LDH release in the medium. As depicted in Figure 3E, cells transfected with TDP-43 4 µg presented an increase in the LDH released in the medium compared to the control cells. To further demonstrate TDP-43 ability to decrease cell viability, transfected cells were incubated with PI. Flow cytometry analysis confirmed these results and showed an increase in cellular death caused by TDP-43 at 3 and 4 µg (*p* < 0.05; Figure 3F). The microscopic examination of cells transfected with increasing concentrations of TDP-43 demonstrated cells with irregular cell outlines, decreased cell density, and increased number of detached cells. These alterations in cellular morphology were accompanied by an increase in the number of PI-positive cells (Figure 3, panel G). On the other hand, transfection of HEK293T cells with the highest plasmid concentration coding for two control non-toxic proteins (PTCHD1 and SOD1), did not show any alterations in cell viability (Figures S2B,C and S3).

### 3.3. Oxidative Stress and Energy Metabolism Evaluation

**Figure 4:**
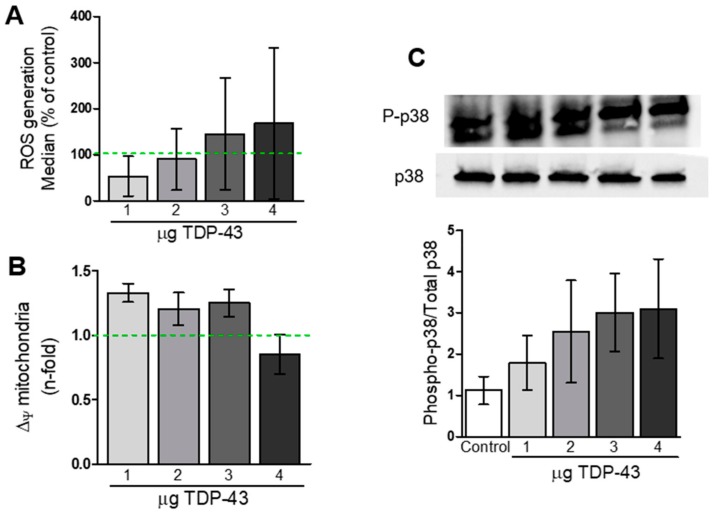
Analysis of TDP-43 overexpression, oxidative stress, and impairment in mitochondrial membrane potential in HEK293T cells. (**A**) Median fluorescence of DCF-DA signal from TDP-43-transfected cells in relation to the control. Statistical analysis revealed no differences between groups. (**B**) Membrane mitochondrial potential analyzed with TMRM probe revealed no alterations induced by TDP-43 overexpression (*n* = 3–8). (**C**) Western blot analysis of p38 phosphorylation in whole-cell lysates from TDP-43-overexpressing cells (*n* = 4–5).

Flow cytometry analysis of cells incubated with H2DCF-DA suggested a trend towards increase in ROS production in TDP-43 overexpressing cells, but statistical analysis revealed no differences between groups (Figure 4A).

We also investigated if TDP-43 overexpression could induce mitochondrial impairment in HEK293T cells. Flow cytometry analysis revealed a trend to an increase in the percentage of cells with depolarized mitochondrial membranes (∆_Ψ_) in almost all TDP-43 conditions, but no statistical differences were found (Figure 4B).

Knowing that the p38 mitogen-activated protein kinase (MAPK) pathway is usually activated in cases of cell apoptosis, we decided to investigate if TDP-43 overexpression could alter the activation of p38 pathway. Western blot analysis revealed a trend of TDP-43 to induce increase in the ratio Phosphorylated-p38/Total p38 MAPK, although no statistical significance was observed between groups (Figure 4C).

**Figure 5:**
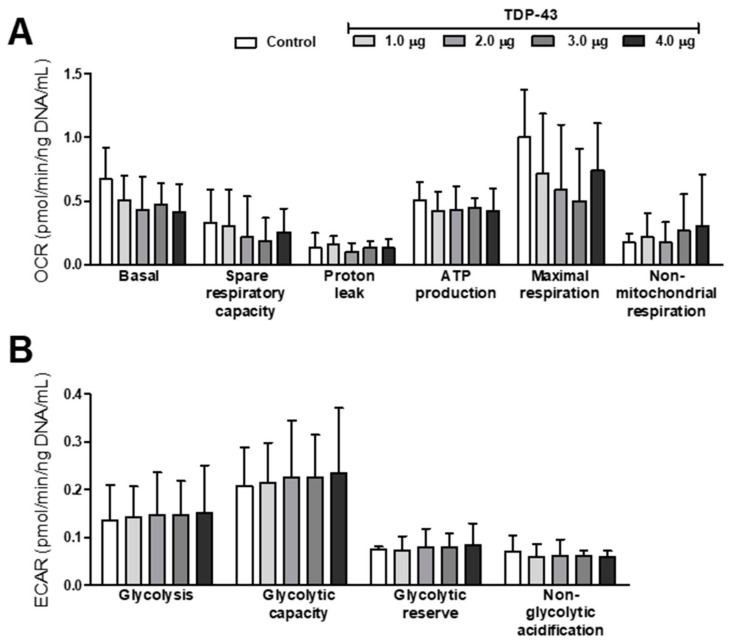
TDP-43 overexpression induces dose-dependent trends of modifications in energy metabolism. (**A**) Oxygen consumption rate (OCR); (**B**) Extracellular acidification rate (ECAR). Data are presented as mean ± SEM. Statistical analysis revealed no differences between groups (Kruskal–Wallis nonparametric test; *n* = 3–4).

Analyses of energy metabolism, that is, oxidative phosphorylation (trough Oxygen Consumption Rate; OCR) and glycolysis (trough ExtraCellular Acidification Rate; ECAR), revealed a tendency of dose–response effect of TDP-43 overexpression. Data in Figure 5 suggest a decrease in basal and maximal respiration and an increase in non-mitochondrial respiration, glycolysis, and glycolytic capacities (Figure 5A,B), but statistical analysis showed no differences between groups. The ratio of OCR max and ECAR max suggests the occurrence of the Warburg effect, but we found no statistical differences between control and TDP-43-overexpressing cells (data not shown).

### 3.4. Metabolome Profile

**Figure 6:**
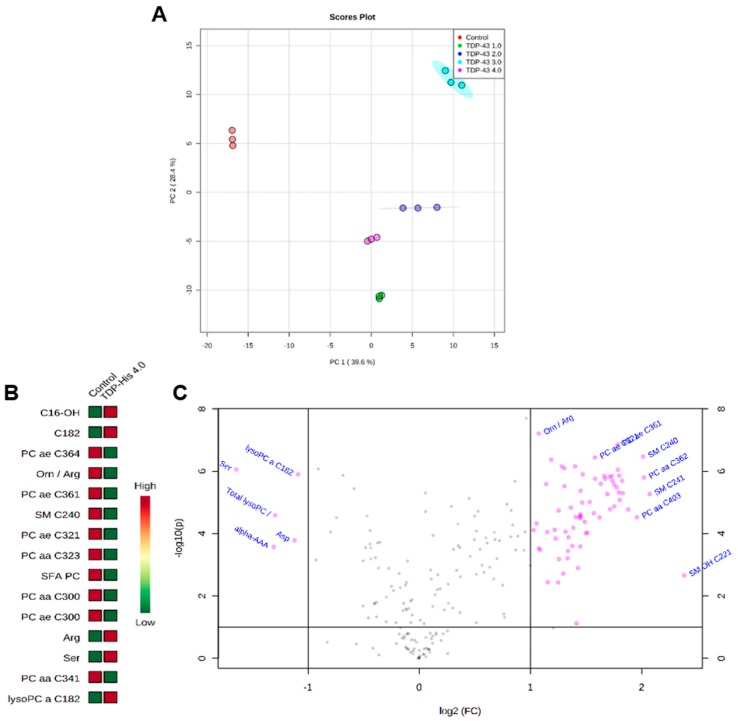
(**A**) Metabolomics analyses reveal a set of distinguishable metabolites altered in TDP-43-overexpressing HEK293T cells. (**B**,**C**) VIP metabolites and volcano plot analysis are represented for TDP-43 4 µg. Metabolomics analyses were realized with MetaboAnalyst (http://www.metaboanalyst.ca).

PLS-DA scatter plot showed an excellent intragroup variability and discrimination of the four groups (all separated) with a clear separation of the control group through the first component (Figure 6A; Principal Component 1: 39.6%; Principal Component 2: 28.4%). Further analysis of the most discriminant metabolites identified by both VIP values > 1 and by fold-change > 2 in the TDP-43 4µg condition revealed a decrease in the levels of glycerophospholipids (like PC ae C30:0, PC ae C32:1, PC ae C36:1, PC ae C36:4, PC aa C30:0, PC aa C32:3, and PC aa C34:1), sphingolipid (SM C24:0), in the ratio Ornithine/Arginine (an indicator of Arginase activity) and in the sum of saturated glycerophosphocholines (SFA PC). On the other hand, we found an increase in the levels of the lysophosphatidylcholine C18:2 (lysoPC a C18:2) and serine in the metabolome of cells transfected with TDP-43 4 µg (Figure 6B,C).

Interestingly, analysis of the metabolites commonly altered in all concentrations of TDP-43 tested revealed alterations in levels of mono-unsaturated fatty acids (MUFA), poly-unsaturated fatty acids (PUFA), and saturated fatty acids, among others (Table S1). These findings further link TDP-43 overexpression with alterations on cell metabolism.

## 4. Discussion

In the present study, we showed that TDP-43 overexpression in HEK293T cells with different concentrations of TDP-43 plasmid induce alterations in cell function, expression in the cytoplasm, and aggregation in a concentration-dependent way. Further, it induces expression of cleaved TDP-43 forms as found in ALS patients, namely 25- and 35-kDa fragments. Transfection of HEK293T cells (250.000 cells) one day after plating with 4 µg of TDP-43-encoding plasmid induced a decrease in cell viability. This feature was shown by diverse complementary findings, as reduction in the number of alive, attached cells, increase in cells arrested at G2/M phase, and increase in nuclear fragmentation (sub-G1 events). TDP-43 overexpression altered cells’ morphology in a dose-dependent way. Interestingly, cells transfected with both 3 µg and 4 µg of TDP-43-enconding plasmid showed a decrease in MTT reduction and increase in PI staining, while TDP-43 4 µg increased the LDH released into the medium. TDP-43 overexpression did not alter mitochondrial membrane potential nor ROS levels. Evaluation of mitochondrial metabolism suggested a concentration-dependent tendency of TDP-43 in decreasing basal and maximal respiration followed by increased non-mitochondrial respiration, glycolysis, and glycolytic capacity. As demonstrated by metabolomics analysis, TDP-43 4 µg presents the most discriminant profile of metabolites when compared to control group and the other TDP-43 concentrations tested.

### 4.1. TDP-43 Overexpression Induces Protein Aggregation and Fragmentation.

In our cellular model, the overexpression of TDP-43 revealed common characteristics observed in ALS patients as the presence of CTFs with 25- and 35-kDa. Although there is controversy regarding whether TDP-43 is overexpressed in ALS patients [13], the presence of CTFs could represent a consequence of loss in protein homeostasis that leads to protein accumulation. This is supported by the finding that TDP-43 accumulation in the cytoplasm deregulates autophagy and proteasome functions [14,15,16]. Further, the presence of CTFs could represent a cellular way to cope with the increase in protein accumulation, by cleaving it in smaller pieces that could more easily enter the ubiquitin–proteasome system (UPS) and autophagy systems [17,18].

### 4.2. Decrease in Cellular Viability Induced by TDP-43 Overexpression

Alteration in cell viability associated with TDP-43 was shown here in several complementary protocols, from reduction in the number of alive cells, decrease in the capacity of cells to metabolize MTT, increased release of LDH into the medium, and increase in cells permeant to PI. The similar profile of reduced cellular viability was demonstrated here by different protocols: (1) strongly confirmed the effect of TDP-43 in decreasing cell viability and (2) broadened the number of techniques researchers can apply, adapted to the material available (for example, an absorbance plate reader for MTT and LDH test, or a flow cytometer for PI staining). Microscopic examination of cells transfected with increasing concentrations of TDP-43 also evidenced the toxicity caused by TDP-43 overexpression in a concentration-dependent way. Intriguingly, transfection with 2 µg or 3 µg of TDP-43-encoding plasmid induced a decrease in the number of cells arrested in S phase, but only the condition of TDP-43 3µg was followed by a decrease in cell viability. The increase in the number of cells arrested in the G2/M phase was followed by an increase in nuclear fragmentation when cells were transfected with the higher concentration of TDP-43 tested here, meaning that TDP-43 at 4 µg inhibited cellular proliferation that then entered apoptosis pathways. These results could also explain the alterations in cellular morphology and the decreased number of adherent cells when analyzing cells under the microscope. Our findings are in accordance with a previous study performed in SH-SY5Y cells [19]. However, in the vast majority of studies regarding TDP-43-overexpression-mediated toxicity, information about the quantity of plated cells, as well as concentration of cDNA used to transfect cells, is missing. This information is of utmost importance to allow researchers to reproduce findings about TDP-43 toxicity worldwide.

Activation of p38 MAPK pathway is known to occur in cases of cellular stress and DNA damage. p38 MAPK pathway may markedly contribute to neurodegenerative disorders, including Parkinson’s and Alzheimer’s disease and ALS [20]. In ALS mouse models, p38 MAPK phosphorylation increases in motor neurons of the spinal cord before the emergence of clinical symptoms [21,22]. In the present study, we showed a trend of increased p38 activation induced by TDP-43 overexpression, which could be related to the cell cycle arrest and apoptosis induced by TDP-43.

### 4.3. TDP-43 Overexpression and Mitochondrial Function

Our data presented here suggested that overexpression of TDP-43 can induce an alteration in mitochondrial membrane polarization, but further analysis revealed no statistical differences between TDP-43-overexpressing cells and control cells. We also observed a tendency of alterations in the oxidative phosphorylation and glycolysis capacities of cells overexpressing TDP-43, in a concentration-dependent way. Again, no significant differences could be observed. Despite our data, impaired mitochondrial function was shown to be closely related to ALS pathophysiology (Muyderman and Chen, 2014). Mitochondrial dysfunction is another toxic effect mediated by TDP-43 according to independent reports [23,24], and TDP-43 colocalization in the mitochondria seems to be essential for this deleterious effect [25,26].

Although mitochondrial impairment is closely related to an increase in the levels of ROS, and several reports already demonstrated that TDP-43 induces increase in the ROS levels, our results do not support such findings. These discrepancies could be due to technical reasons, since our results showed large variances, or to the fact that there are several ways to measure oxidative stress. For example, Wang et al. (2019) use the fluorescent probe mitoSOX (which specifically measures ROS levels in the mitochondria) and fixate cells [23]. Other studies focus on the effect of oxidative stress in inducing TDP-43 aggregation [27,28] and the fact that this might occur in a feedforward manner via activation of AMPK [29,30,31,32]. In our model, after 48 h of transfection, TDP-43 did not induce significant mitochondrial impairment nor oxidative stress. Further studies are needed to confirm these results.

### 4.4. Alteration of Cell Metabolome Induced by TDP-43 Overexpression

We also realized metabolomics analyses to investigate the effect of TDP-43 overexpression over the cell metabolome to evaluate if these alterations reflect the alterations found by metabolomics studies performed in ALS patients [33]. Although ALS is primarily a neurological disease, it also affects diverse nonneural cell types. For example, pathological mechanisms associated with ALS have been observed in muscle cells [34] and skin fibroblasts [35]. Here, we focused our metabolomics analysis in the condition TDP-43 4 µg, which is highly discriminated from controls. Interestingly, we found a variety of glycerophospholipids decreased in this condition. Such a decrease was already shown in a SOD1-mouse model of ALS and could be related to the neuronal loss observed in these animals [36]. One possible hypothesis is that TDP-43 alters the composition of the cellular membrane and renders it more permeable (following the membrane hypothesis of aging [37]). The increase in the permeability of the cell membrane induced by TDP-43 can also explain the increase in PI staining at TDP-43 4 µg. However, more experiments are needed to confirm this hypothesis—as, for example, performing metabolomics analysis in isolated cell membrane. Moreover, all concentrations of TDP-43 were linked with alterations in levels of PUFAs. Metabolomics studies also reported alterations in PUFA levels in ALS patients [38]. This is the first time that metabolomics alterations induced by TDP-43-overexpression in a human cell line are reported. Our findings are in agreement with those from ALS patients and a recognized ALS mouse model (the SOD1-G93A), supporting our model as a cellular model that reproduces interesting findings from ALS patients.

**Figure 7:**
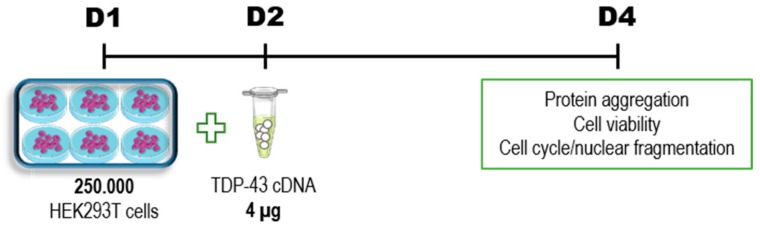
A fast, reproducible and reliable protocol for assessment of TDP-43-associated toxicity. Our protocol reveals the best markers of toxicity associated with TDP-43 overexpression: evaluation of protein aggregation (analyzed by western blot in differential cell lysates), cell viability (by MTT reduction, LDH release, and PI incorporation), and cell cycle assessment (after cell lysis and PI incorporation). These parameters should be employed for the evaluation of possible therapeutic agents against TDP-43-mediated toxicity and possible application as ALS therapeutics.

While most studies focus on mutations in the TDP-43 protein, in the present study we highlight the toxic role of the wild type, full-length form of TDP-43 protein—the most common in ALS cytoplasmic aggregates. Here, we provided a fast and reproducible protocol for researchers to evaluate potential therapeutic candidates against TDP-43-mediated toxicity by means of protein localization and aggregation, cell viability (MTT reduction, LDH release, and PI staining), and cell cycle arrest (Figure 7). Further, we emphasized the reliability of overexpression studies to recapitulate findings from ALS patients. We suggest that this protocol will be useful for researchers worldwide in the search for new therapeutic options against ALS, with a more focused approach targeting TDP-43-associated toxicity. Identification of therapeutic agents with the potential to decrease TDP-43 cytoplasmic aggregation could halt TDP-43-associated toxicity and the consequent neurodegeneration, slowing down the relentless progression of the disease observed in ALS patients.

## Supplementary Materials

The following are available online at https://www.mdpi.com/2073-4409/9/1/68/s1. Figure S1: Representative graphs of flow cytometry analysis (FlowJo VX software). Figure S2: A. HEK293T cells transfected with increasing concentrations of plasmids encoding GFP or GFP-TDP-43 proteins show a distinct pattern of decreased cell viability. Figure S3: Evaluation of cell viability alteration induced by overexpression of a control protein, PTCHD1, transfected at 4 µg.

## References

[1] Buratti E., Baralle F.E. (2001). Characterization and functional implications of the RNA binding properties of nuclear factor TDP-43, a novel splicing regulator of CFTR exon 9. J. Biol. Chem..

[2] Polymenidou M., Lagier-Tourenne C., Hutt K.R., Huelga S.C., Moran J., Liang T.Y., Ling S.C., Sun E., Wancewicz E., Mazur C. (2011). Long pre-MRNA depletion and RNA missplicing contribute to neuronal vulnerability from loss of TDP-43. Nat. Neurosci..

[3] Hergesheimer R.C., Chami A.A., de Assis D.R., Vourc’h P., Andres C.R., Corcia P., Lanznaster D., Blasco H. (2019). The debated toxic role of aggregated TDP-43 in amyotrophic lateral sclerosis: A resolution in sight?. Brain J. Neurol..

[4] Baralle M., Buratti E., Baralle F.E. (2013). The role of TDP-43 in the pathogenesis of ALS and FTLD. Biochem. Soc. Trans..

[5] Prasad A., Bharathi V., Sivalingam V., Girdhar A., Patel B.K. (2019). Molecular mechanisms of TDP-43 misfolding and pathology in amyotrophic lateral sclerosis. Front. Mol. Neurosci..

[6] Crowley L.C., Chojnowski G., Waterhouse N.J. (2016). Measuring the DNA content of cells in apoptosis and at different cell-cycle stages by propidium iodide staining and flow cytometry. Cold Spring Harb. Protoc..

[7] Patin F., Baranek T., Vourc’h P., Nadal-Desbarats L., Goossens J.F., Marouillat S., Dessein A.F., Descat A., Hounoum B.M., Bruno C. (2016). Combined metabolomics and transcriptomics approaches to assess the IL-6 blockade as a therapeutic of ALS: Deleterious alteration of lipid metabolism. Neurother. J. Am. Soc. Exp. NeuroTher..

[8] Eskelinen M.H., Kivipelto M. (2010). Caffeine as a protective factor in dementia and alzheimer’s disease. J. Alzheimers Dis. JAD.

[9] Xia J., Wishart D.S. (2016). Using metaboanalyst 3.0 for comprehensive metabolomics data analysis. Curr. Protoc. Bioinform..

[10] Madji Hounoum B., Blasco H., Coque E., Vourc’h P., Emond P., Corcia P., Andres C.R., Raoul C., Mavel S. (2018). The metabolic disturbances of motoneurons exposed to glutamate. Mol. Neurobiol..

[11] Ganini D., Leinisch F., Kumar A., Jiang J., Tokar E.J., Malone C.C., Petrovich R.M., Mason R.P. (2017). Fluorescent proteins such as eGFP lead to catalytic oxidative stress in cells. Redox Biol..

[12] Ansari A.M., Ahmed A.K., Matsangos A.E., Lay F., Born L.J., Marti G., Harmon J.W., Sun Z. (2016). Cellular GFP toxicity and immunogenicity: Potential confounders in In Vivo cell tracking experiments. Stem Cell. Rev. Rep..

[13] Swarup V., Phaneuf D., Dupre N., Petri S., Strong M., Kriz J., Julien J.P. (2011). Deregulation of TDP-43 in amyotrophic lateral sclerosis triggers nuclear factor kappaB-mediated pathogenic pathways. J. Exp. Med..

[14] Leibiger C., Deisel J., Aufschnaiter A., Ambros S., Tereshchenko M., Verheijen B.M., Buttner S., Braun R.J. (2018). TDP-43 controls lysosomal pathways thereby determining its own clearance and cytotoxicity. Hum. Mol. Genet..

[15] Budini M., Buratti E., Morselli E., Criollo A. (2017). Autophagy and its impact on neurodegenerative diseases: New roles for TDP-43 and C9orf72. Front. Mol. Neurosci..

[16] Cascella R., Fani G., Capitini C., Rusmini P., Poletti A., Cecchi C., Chiti F. (2017). Quantitative assessment of the degradation of aggregated TDP-43 mediated by the ubiquitin proteasome system and macroautophagy. FASEB J..

[17] Huang C.C., Bose J.K., Majumder P., Lee K.H., Huang J.T., Huang J.K., Shen C.K. (2014). Metabolism and mis-metabolism of the neuropathological signature protein TDP-43. J. Cell Sci..

[18] Li Q., Yokoshi M., Okada H., Kawahara Y. (2015). The cleavage pattern of TDP-43 determines its rate of clearance and cytotoxicity. Nat. Commun..

[19] Yamashita M., Nonaka T., Hirai S., Miwa A., Okado H., Arai T., Hosokawa M., Akiyama H., Hasegawa M. (2014). Distinct pathways leading to TDP-43-induced cellular dysfunctions. Hum. Mol. Genet..

[20] Kim E.K., Choi E.J. (2015). Compromised MAPK signaling in human diseases: An update. Arch. Toxicol..

[21] Tortarolo M., Veglianese P., Calvaresi N., Botturi A., Rossi C., Giorgini A., Migheli A., Bendotti C. (2003). Persistent activation of p38 mitogen-activated protein kinase in a mouse model of familial amyotrophic lateral sclerosis correlates with disease progression. Mol. Cell. Neurosci..

[22] Wengenack T.M., Holasek S.S., Montano C.M., Gregor D., Curran G.L., Poduslo J.F. (2004). Activation of programmed cell death markers in ventral horn motor neurons during early presymptomatic stages of amyotrophic lateral sclerosis in a transgenic mouse model. Brain Res..

[23] Wang P., Deng J., Dong J., Liu J., Bigio E.H., Mesulam M., Wang T., Sun L., Wang L., Lee A.Y. (2019). TDP-43 induces mitochondrial damage and activates the mitochondrial unfolded protein response. PLoS Genet..

[24] Gautam M., Jara J.H., Kocak N., Rylaarsdam L.E., Kim K.D., Bigio E.H., Hande Ozdinler P. (2019). Mitochondria, ER, and nuclear membrane defects reveal early mechanisms for upper motor neuron vulnerability with respect to TDP-43 pathology. Acta Neuropathol..

[25] Davis S.A., Itaman S., Khalid-Janney C.M., Sherard J.A., Dowell J.A., Cairns N.J., Gitcho M.A. (2018). TDP-43 interacts with mitochondrial proteins critical for mitophagy and mitochondrial dynamics. Neurosci. Lett..

[26] Salvatori I., Ferri A., Scaricamazza S., Giovannelli I., Serrano A., Rossi S., D’Ambrosi N., Cozzolino M., Giulio A.D., Moreno S. (2018). Differential toxicity of TAR DNA-Binding protein 43 isoforms depends on their submitochondrial localization in neuronal cells. J. Neurochem..

[27] Meyerowitz J., Parker S.J., Vella L.J., Ng D., Price K.A., Liddell J.R., Caragounis A., Li Q.X., Masters C.L., Nonaka T. (2011). C-Jun N-terminal kinase controls TDP-43 accumulation in stress granules induced by oxidative stress. Mol. Neurodegener..

[28] Iguchi Y., Katsuno M., Takagi S., Ishigaki S., Niwa J., Hasegawa M., Tanaka F., Sobue G. (2012). Oxidative stress induced by glutathione depletion reproduces pathological modifications of TDP-43 linked to TDP-43 proteinopathies. Neurobiol. Dis..

[29] Ayala V., Granado-Serrano A.B., Cacabelos D., Naudi A., Ilieva E.V., Boada J., Caraballo-Miralles V., Llado J., Ferrer I., Pamplona R. (2011). Cell stress induces TDP-43 pathological changes associated with ERK1/2 dysfunction: Implications in ALS. Acta Neuropathol..

[30] Dewey C.M., Cenik B., Sephton C.F., Dries D.R., Mayer P., Good S.K., Johnson B.A., Herz J., Yu G. (2011). TDP-43 is directed to stress granules by sorbitol, a novel physiological osmotic and oxidative stressor. Mol. Cell. Biol..

[31] D’Amico E., Factor-Litvak P., Santella R.M., Mitsumoto H. (2013). Clinical perspective on oxidative stress in sporadic amyotrophic lateral sclerosis. Free Radic. Biol. Med..

[32] Liu Y.J., Lee L.M., Lai H.L., Chern Y. (2015). Aberrant activation of AMP-activated protein kinase contributes to the abnormal distribution of HuR in amyotrophic lateral sclerosis. FEBS Lett..

[33] Lanznaster D., de Assis D.R., Corcia P., Pradat P.F., Blasco H. (2018). Metabolomics Biomarkers: A Strategy Toward Therapeutics Improvement in ALS. Front. Neurol..

[34] Wong M., Martin L.J. (2010). Skeletal muscle-restricted expression of human SOD1 causes motor neuron degeneration in transgenic mice. Hum. Mol. Genet..

[35] Yang S., Zhang K.Y., Kariawasam R., Bax M., Fifita J.A., Ooi L., Yerbury J.J., Nicholson G.A., Blair I.P. (2015). Evaluation of skin fibroblasts from amyotrophic lateral sclerosis patients for the rapid study of pathological features. Neurotox. Res..

[36] Arima H., Omura T., Hayasaka T., Masaki N., Hanada M., Xu D., Banno T., Kobayashi K., Takeuchi H., Kadomatsu K. (2015). Reductions of docosahexaenoic acid-containing phosphatidylcholine levels in the anterior horn of an ALS mouse model. Neuroscience.

[37] Yu Q., Zhong C. (2018). Membrane Aging as the real culprit of alzheimer’s disease: Modification of a hypothesis. Neurosci. Bull..

[38] Nagase M., Yamamoto Y., Miyazaki Y., Yoshino H. (2016). Increased oxidative stress in patients with amyotrophic lateral sclerosis and the effect of edaravone administration. Redox Rep. Commun. Free Radic. Res..

