# Supplementary material for: TDP-43-Mediated Toxicity in HEK293T Cells: A Fast and Reproducible Protocol To Be Employed in the Search of New Therapeutic Options against Amyotrophic Lateral Sclerosis
Source: Cells. 2019 Dec 26;9(1):68. doi: 10.3390/cells9010068 (PMC7016571; doi:10.3390/cells9010068)
Supplement: Supplementary file 1 [file cells-09-00068-s001.pdf]

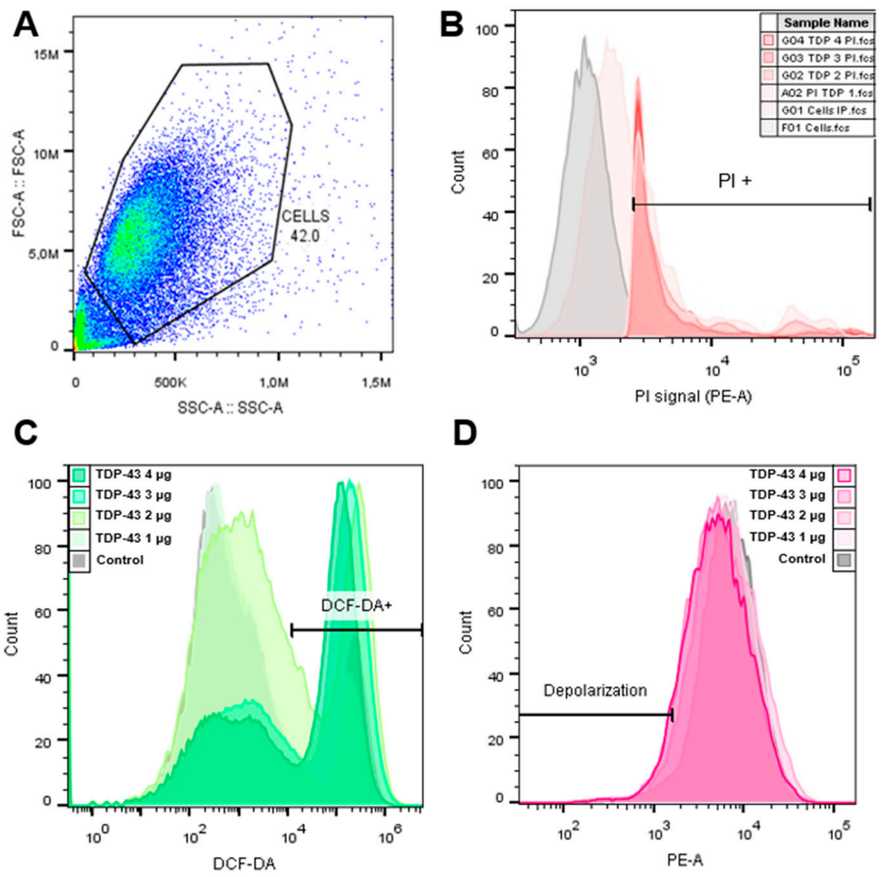

Figure S1. Representative graphs of flow cytometry analysis (FlowJo VX software). (**A**) Alive HEK293T cells used for analysis. (**B**) Representative graph for PI positive signal. (**C**) Representative graph for DCF-DA signal. (**D**) Representative graph for TMRM signal.

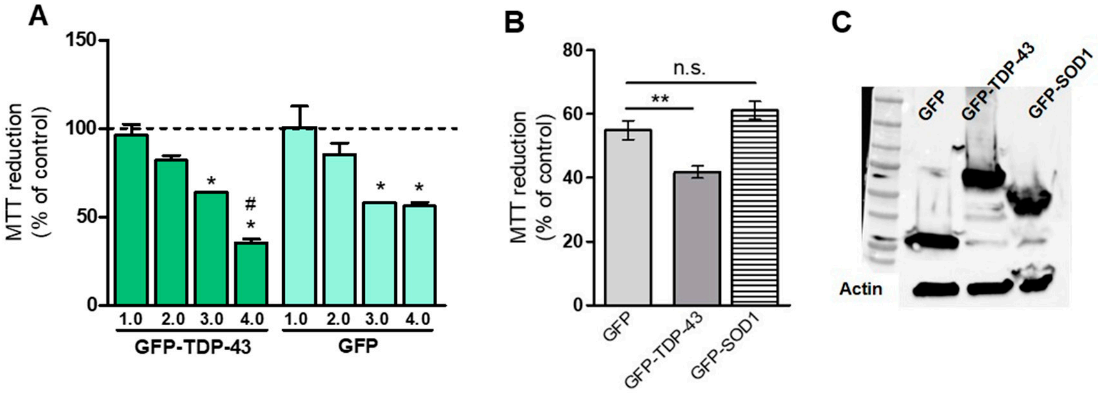

Figure S2. A. HEK293T cells transfected with increasing concentrations of plasmids encoding GFP or GFP-TDP-43 proteins show a distinct pattern of decreased cell viability. n = 3–4; Nonparametric Kruskal-Wallis test: *$p < 0.05$ compared to the control group (hatched bar); #$p < 0.01$ (Mann-Whitney test) between GFP-TDP-43 4 μg and GFP 4 μg. B. SOD1-GFP, used as a cytoplasmic control protein, induce the same decrease in cell viability as GFP alone. Cells transfected with GFP-TDP-43 exhibit a higher decrease in cell viability compared to both GFP and GFP-SOD1. All plasmids transfected at 4 μg. n = 3. Nonparametric Kruskal-Wallis test: **$p < 0.01$; n.s.: non significant. C. GFP, GFP-TDP-43 and GFP-SOD1 protein are expressed in the same amount by HEK293T cells.

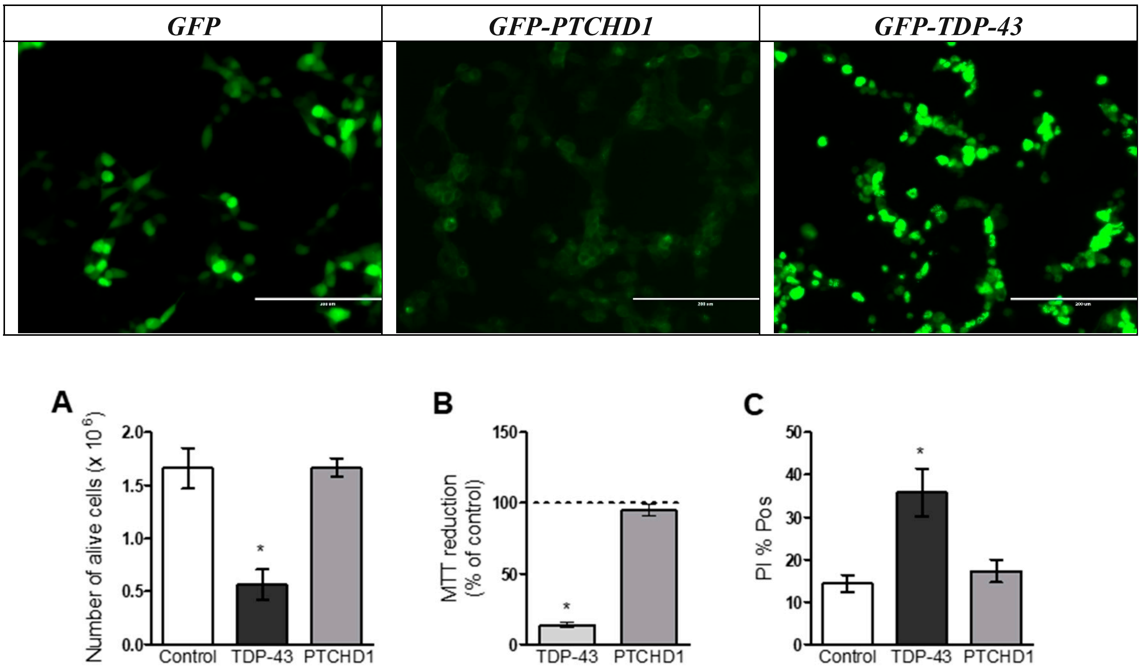

Figure S3. Evaluation of cell viability alteration induced by overexpression of a control protein, PTCHD1, transfected at 4 μg. **Top:** Visualization of GFP, GFP-PTCHD1 and GFP-TDP-43 expression in HEK293T cells transfected with 4μg of each plasmid. **Bottom:** Cells were transfected with 4μg of plasmids pcDNA3.3 (control), TDP-43-6His or PTCHD1. (**A**) Number of viable cells after Trypan test exclusion. (**B**) MTT reduction test. (**C**) Incorporation of Propidium Iodide (PI) test. Non-parametric Kruskal-Wallis test revealed $p < 0.05$ compared to control groups (hatched bar in graph B). n = 3.

Table S1. List of metabolites found altered in all TDP-43 conditions.

Mono-unsaturated fatty acids (MUFA)	
Poly-unsaturated fatty acid (PUFA)	
Saturated fatty acids (SFA)	
Glycerophospholypids	lysoPC a C26:1
	lysoPC a C28:1
	PC aa C28:1
	PC aa C30:0
	PC aa C32:0
	PC aa C32:1
	PC aa C32:2
	PC aa C32:3
	PC aa C34:1
	PC aa C34:2
	PC aa C34:3
	PC aa C36:1
	PC aa C36:2
	PC aa C36:3
	PC aa C38:3
	PC aa C40:3
	PC aa C40:5
	PC ae C30:1
	PC ae C32:1
	PC ae C32:2
	PC ae C34:0
	PC ae C34:1
	PC ae C34:2
	PC ae C34:3
	PC ae C36:1
	PC ae C36:2
	PC ae C36:3
	PC ae C38:2
	PC ae C38:3
	PC ae C38:5
	PC ae C40:3
Ratio Total lysoPC/Total PC	
Total phosphatidylcholines	Diacyl-PC
	Acyl-alkyl-PC
Total phosphatidylcholines-sphingolipids	
Sphingolipids	SM C16:0
	SM C18:0
	SM C18:1
	SM C24:0
	SM OH C14:1
	SM OH C16:1
	SM OH C22:1
	SM OH C22:2
Total sphingomyeline (SM)	
Total SM-non OH	

PC: phosphatidylcholines; lysoPC: lyso-phosphatidylcholines.
